# Novel two-chain structure utilizing KIRS2/DAP12 domain improves the safety and efficacy of CAR-T cells in adults with r/r B-ALL

**DOI:** 10.1016/j.omto.2021.08.014

**Published:** 2021-08-28

**Authors:** Ming Sun, Peipei Xu, Enxiu Wang, Min Zhou, Tongpeng Xu, Jing Wang, Qian Wang, Bo Wang, Kaihua Lu, Chen Wang, Bing Chen

**Affiliations:** 1 Department of Hematology, Nanjing Drum Tower Hospital, The Affiliated Hospital of Nanjing University Medical School, Nanjing 210008, PR China; 2 Department of Oncology Center, The Affiliated Suzhou Hospital of Nanjing Medical University, Suzhou Municipal Hospital, Gusu School, Suzhou, PR China; 3 Nanjing CART Medical Technology Co., Ltd., Nanjing 210032, PR China; 4 Department of Pathology, The Affiliated Hospital of Youjiang Medical University for Nationalities, Baise 533000, PR China; 5 Clinical Pathological Diagnosis & Research Center, Youjiang Medical University for Nationalities, Baise 533000, PR China; 6 The Key Laboratory of Molecular Pathology (Hepatobiliary Diseases) of Guangxi, Baise 533000, PR China; 7 Department of Oncology, First Affiliated Hospital, Nanjing Medical University, Nanjing, PR China; 8 Jiangsu Runtian Pharmaceutical Chain Pharmacy Co., Ltd., Nanjing 210000, PR China; 9 Department of Medical Oncology, The Seventh Affiliated Hospital, Sun Yat-Sen University, Shenzhen 518107, PR China; 10 Department of Research and Development, Nanjing Aide Institute of Immunotherapy, Nanjing 211808, PR China; 11 Clinical College of Traditional Chinese and Western Medicine, Nanjing University of Chinese Medicine, Nnajing 210008, PR China

**Keywords:** r/r B-ALL, CAR-T cells, KIRS2/DAP12 signaling domain, 4-1BB, clinical trial

## Abstract

Engineered T cells that express chimeric antigen receptors (CARs) have been a promising therapy for hematologic malignancies. The optimization of CAR structure using different signaling domains can alter a wide range of CAR-T cell properties, including anti-tumor activity, long-term persistence, and safety. In this study, we developed a novel CAR structure based on KIRS2/Dap12 for B cell acute lymphoblastic leukemia (B-ALL) antigen CD19 and compared the anti-tumor efficacy and safety of this construct in transduced T cells with standard second-generation CAR-T cells targeting CD19 for B-ALL *in vitro* and *in vivo* and in adult relapsed/refractory (r/r) B-ALL patients. We discovered that KIRS2/Dap12 receptor infused with 4-1BB co-stimulation domain could enhance anti-tumor efficacy by remarkably increasing the production of pro-inflammatory interleukin-2 (IL-2), especially when co-cultured with antigen-positive tumor cells. In addition, CD19-KIRS2/Dap12-BB CAR-T cells showed the inspiring outcome that complete responses were seen in 4 of 4 (100%) patients without neurotoxicity and a high rate of severe cytokine release syndrome (CRS) after CAR-T infusion in a phase I clinical trial. Given these encouraging findings, CD19-KIRS2/Dap12-BB CAR-T cells are safe and can lead to clinical responses in adult patients with r/r B-ALL, indicating that further assessment of this therapy is warranted.

## Graphical abstract

**Figure:**
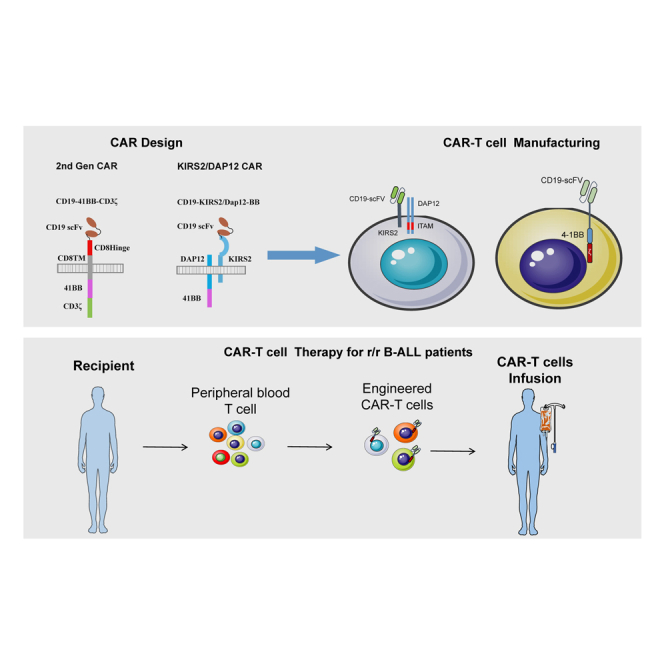

## Teaser

Recently, CD19-targeted CAR-T cells have been approved by the FDA for hematologic malignancy therapy and achieve outstanding clinical outcome. Chen et al.’s findings indicate that r/r B-ALL patients can benefit from the KIRS2/Dap12-BB CAR-T cells in the future.

## Introduction

Adoptive T cell therapy (ACT) involves the manufacture of a patient’s T cells followed by infusion of these engineered T cells into the patient with cancer.^1^^,^^2^ In recent years, chimeric antigen receptor (CAR) T cell immunotherapy, based on the infusion of engineered autologous T cells to recognize the tumor-associated antigens expressed on cancer cells, has changed the modality of treatment for hematological malignancies.^3^^,^^4^ Specifically, CAR-T cells targeting CD19 or B cell maturation antigen (BCMA) in treating B cell lymphoma, leukemia, and multiple myeloma have achieved unprecedented response rates.^5^^,^^6^ Synonymous with the outstanding clinical outcome of CAR-T cell therapy in hematological malignancies have been severe toxicities, including cytokine-release syndrome (CRS), neurotoxicity syndrome, CAR-T cell-related encephalopathy syndrome (CRES), and hemophagocytic lymphohistiocytosis/macrophage activation syndrome (HLH/MAS).^7^ Therefore, management of these toxicities has been a major concern for clinical implementation.

A growing amount of evidence indicates that CAR toxicity may be linked to the synthetic nature of the receptor design.^8^ To improve the safety of CAR-transduced T cells, we previously designed a natural multi-chain immunoreceptor CAR based on the DNAX-activating protein of 12 kDa (Dap12) signaling domain for the first time, which triggers antigen-specific cytotoxicity, cytokine production, and proliferation that is comparable with CD3z-based CARs *ex vivo*/*in vitro* for hematological malignancies.^9^^,^^10^ Dap12 is a transmembrane signaling adaptor protein containing a single immunoreceptor tyrosine-based activation motif (ITAM) that has low homology with ITAMs identified in the CD3z chain. The expression of Dap12 has been found in a variety of immune cells, such as natural killer (NK) cells, macrophages, and some T cells, indicating that Dap12 may have a general role in the immune response.^11^^,^^12^ Dap12 was originally found to activate NK cells when its ligand was ligated with an associated receptor, which then induced the SRC-family kinase activation and phosphorylation of tyrosine residues in the ITAM.^13^^,^^14^ Currently, more than 20 Dap12-related receptors have been uncovered, including TREM1, TREM2, and KIRS et al.

In contrast to the most common CARs utilizing a simplified format to recapitulate the signals necessary for T cell effector function and proliferation, we previously found that T cells expressing a KIRS2/Dap12 CAR showed anti-tumor activity even without the additional domains from costimulatory receptors.^10^ Typically, the second- and third-generation CARs included one or two costimulatory domains derived from CD28 or 4-1BB that associate with the CD3ζ endo-domain.^15^^,^^16^ CD28 signaling is beneficial for T cell cytotoxicity, and 4-1BB/CD3ζ CARs could phosphorylate endogenous CD28 and activated the CD28 signaling pathway.^17^^,^^18^ However, encoding a fully functional CD28 signaling domain on a CAR polypeptide chain may yield excessive stimulation that increases the incidence of CRS, promotes T cell exhaustion, and reduces persistence. Considering the critical importance of co-stimulation for T cell activation and acquisition of effector function, we generated a panel of representative receptors through combining the KIRS2/Dap12 with costimulatory molecule 41-BB in this study. To determine whether they are able to induce a more controlled T cell response and improved T cell activity, we evaluated functional characterization of diverse KIRS2/Dap12 CAR structure and found that KIRS2/Dap12-BB CAR-conferred T cells enhanced antitumor cytotoxicity and increased cytokine production. Furthermore, we present experimental evidence for the efficacy and compatibility of the KIRS2/Dap12-BB platform with different targets in preclinical models of solid and hematological tumors. Finally, a phase I clinical trial using autologous T cells expressing CD19-specific CAR (CD19-KIRS2/Dap12-BB) in adult relapsed/refractory (r/r) B-ALL patients with lymphodepleting chemotherapy was conducted to evaluate the anti-tumor activity and safety of CD19-KIRS2/Dap12-BB CAR-T cells compared with second-generation CAR-T cells targeting CD19.

## Results

### 4-1BB co-stimulation domain confers KIRS2/Dap12 CAR-T cells comparable cytotoxic activity and robust cytokine production

**Figure 1:**
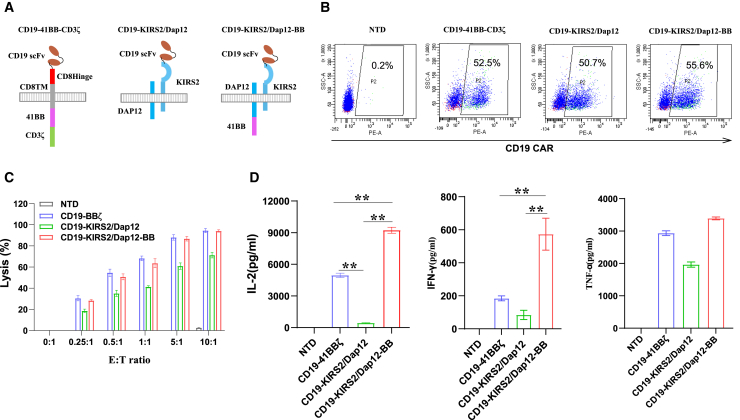
Representative expansion and CAR expression profile of CD19-targeted CAR-T cells (A) Schematic diagram of a CD19-specific CAR. (B) Flow cytometry analysis of the ratio of CAR+ T cells that express anti-mouse FMC63 scFv. (C) Cytotoxicity of CAR-T cells was evaluated by the Real-Time Cell Analyzer (RTCA) system. (D) IFN-γ, TNF-α, and IL-2 released in the culture supernatant by NTD and CAR-T cells were measured by ELISA (n = 3). ∗∗p < 0.01.

Co-stimulation domains in CARs can alter many effector functions of T cells. To directly compare the effector functions induced by 4-1BB co-stimulation, we introduce the signal transduction domains of 4-1BB in KIRS2/Dap12 receptor consisting of the same extracellular domain of anti-CD19 scFv (FMC63) with other CARs (Figure 1A). By using lentiviral vectors and transduction at a multiplicity of infection (2.5), the different CARs could be expressed ranging from 50% to 55% in primary human T cells (Figure 1B). Simultaneously, the CD4/CD8 ratios were also similar (∼20% CD4+ and ∼80% CD8+) (data not shown) among the 3 CAR-expressed T cells. Over 128-fold (population double > 9) expansion of CAR+ T cells could be achieved over the course of transduction and growth in 11 days. To evaluate the anti-tumor cytotoxicity of the 3 types of CAR-T cells, MCF-7 cells that express human CD19 antigen (MCF-7-CD19) were co-cultured with CAR-T cells *in vitro* at different effector:target (E:T) ratios ranging from 0:1 to 10:1 for 20 h. The results showed that these engineered T cells exhibit comparable lytic activity toward MCF-7-CD19 cells (Figure 1C). Furthermore, we observed that T cells expressing 4-1BB domain-infused KIRS2/Dap12 CAR produced greater quantities of interleukin-2 (IL-2) and lower interferon-γ (IFN-γ), and interleukin-6 (IL-6) when compared with cells expressing only KIRS2/Dap12 receptor or 4-1BB/CD3ζ (Figure 1D; Figure S1A). Meanwhile, the results of quantitative PCR (qPCR) analysis also showed that KIRS2/Dap12-BB CAR-T cells express higher IL-2, IFN-γ, granzyme B (GZMB), and IL12A than 4-1BB/CD3ζ CAR-T cells (Figure S1B).

To further assess the primary human T cell differentiation over time, we used a multiparametric flow cytometric approach to distinguish naive-like (Tn, CD45RO−CCR7+), central memory (Tcm, CD45RO+CCR7+), effector memory (Tem, CD45RO+CCR7−), and effector (Teff, CD45RO−CCR7−) T cell subsets. During the expansion phase, the proportions of the Tem subset showed a robust increase from D9 to D11, and KIRS2/Dap12-BB CAR-T cells had higher ratio of Tcm subset than 4-1BB/CD3ζ CAR-T cells (Figure S1C). Moreover, the results of flow cytometric analysis showed that KIRS2/Dap12-BB CAR-T cells had lower PD-1 expression level than 4-1BB/CD3ζ CAR-T cells (Figure S1D).

### 4-1BB infused with Dap12 confers CAR-T cells more IL-2 production

**Figure 2:**
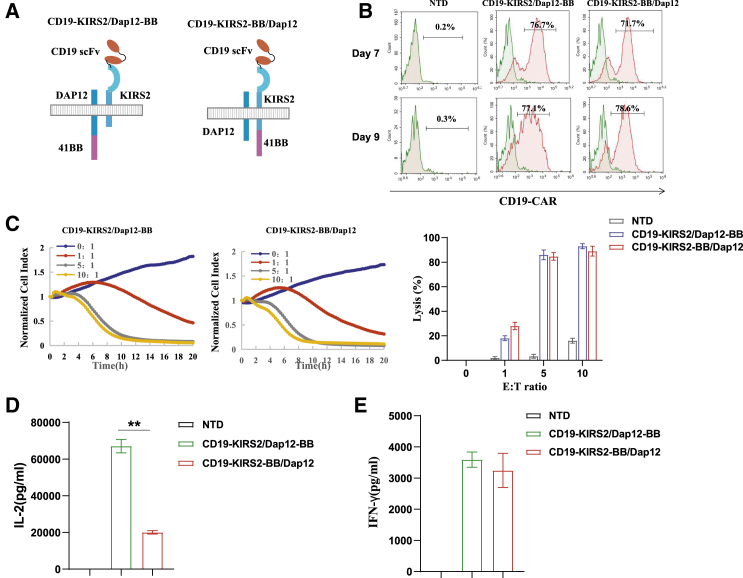
The effect of 4-1BB infusion with different chains on CAR-T cells (A) Schematic diagram of a CD19-specific KIRS2/Dap12 CAR infused with 4-1BB. (B) Flow cytometry analysis of the ratio of CAR+ T cells that express anti-mouse FMC63 scFv. (C) Cytotoxicity of CAR-T cells was evaluated by the RTCA system. (D and E) IFN-γ and IL-2 released in the culture supernatant by NTD and CAR-T cells were measured by ELISA (n = 3). ∗∗p < 0.01.

To explore whether the infusion of 4-1BB with different chains of KIRS2/Dap12 influences the activity and cytokine production of T cells, we generated another CAR in which 4-1BB is infused with the KIRS2 chain and not the Dap12 chain (Figure 2A). The percentage of CAR-positive T cells expressing the two CARS on days 7 and 9 was comparable, with a range from 71% to 78% (Figure 2B). Further cytotoxicity assays showed that 4-1BB infused with different chains exhibits similar lytic activity toward target cells (Figure 2C). Interestingly, 4-1BB infused with the Dap12 chain confers T cells more robust IL-2 production than T cells expressing CAR in which 4-1BB is infused with the KIRS2 chain; however, the production of IFN-γ showed no difference between the two types of CAR-T cells (Figures 2D and 2E).

### KIRS2/Dap12-BB CAR-T cells show efficacy in hematological malignancies and solid tumor models

**Figure 3:**
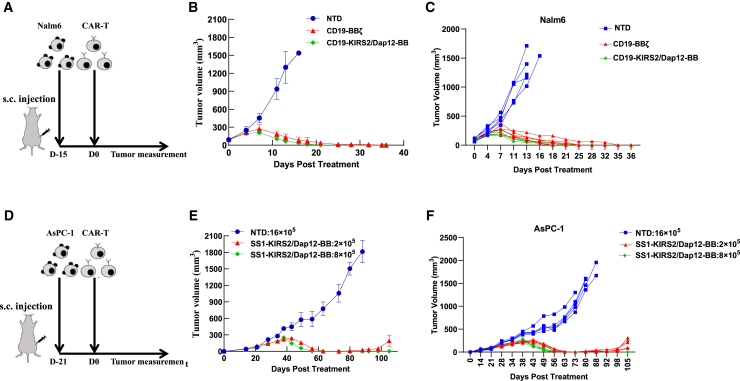
The antitumor activity of CD19- and mesothelin-targeted KIRS2/Dap12 CAR-T cells *in vivo* (A) Schema of the lymphoma xenograft model infused with CAR-T on day 15 after tumor inoculation. (B and C) Nalm6 tumors were subcutaneously (s.c.) established in the right flank of NCG mice and randomly grouped (n = 5 per group). The curve shows the change in tumor volume. (D) Schema of the pancreatic cancer xenograft model infused with CAR-T on day 18 after tumor inoculation. (E and F) AsPC-1 tumors were s.c. established in the right flank of NCG mice and randomly grouped (n = 5 per group). The curve showed the change in tumor volume following treatment with intravenous injections of 2 × 10^5^ or 8 × 10^5^ CAR-T cells.

We next evaluated the anti-tumor cytotoxicity of CD19-KIRS2/Dap12-BB CAR-T cells compared with CAR-T cells engineered with a CD19-targeted 41BB-based second-generation CAR (termed CD19-BBζ CAR) in Nalm6 cell-derived xenograft tumors (Figure 3A). As shown in Figures 3B and 3C, both CD19-KIRS2/Dap12-BB and CD19 BBζ CAR-T cells displayed comparable anti-tumor activity *in vivo*. In striking contrast, untransduced T cells (NTD) showed no anti-tumor activity and tumor progression. Interestingly, analysis of the CD19 CAR-T cells in mouse tail vein blood indicated that KIRS2/Dap12-BB CAR-T cells exhibited stronger proliferative potential and longer persistence than CD19 BBζ CAR-T cells (Figure S1E). These data indicate that CD19-KIRS2/Dap12-BB-modified T cells exhibit control of leukemia as well as CD19-BBz-CAR-T cells, which have been shown to have potent lymphoma activity in humans.

To further determine if the potent anti-tumor activity of T cells bearing a KIRS2/Dap12-CAR is dependent on the CD19 specificity, we generated an additional CAR targeting mesothelin (SS1 KIRS2/Dap12-BB). In an AsPC-1 cell-derived pancreatic adenocarcinoma xenograft model (Figure 3D), T cells expressing SS1-KIRS2/Dap12-BB achieved superior control of tumor growth when compared with untransduced T cells. In this experiment, we established two groups of increasing doses (2 × 10^5^ and 8 × 10^5^ SS1 KIRS2/Dap12-BB CAR-T cells) and found that although pancreatic adenocarcinoma relapsed by day 98 in the low-dose group, the tumor did not relapse for more than 100 days in the high-dose group (Figures 3E and 3F). These findings demonstrate that the activity of KIRS2/Dap12-BB CAR is not antigen dependent, and mesothelin-targeted SS1-KIRS2/Dap12-BB CAR-T cells may have high translation potential anti-tumor activity against mesothelin-positive solid tumors.

### Patient characteristics

Given the encouraging data from the *in vitro* and *in vivo* experiments, we conducted a phase I, single-center, open-label clinical trial (ChiCTR1800016584) to evaluate the feasibility, safety, and clinical and biologic activity of manufacturing and administering CD19-targeted KIRS2/Dap12-BB and BBζ CAR-T cells to adult patients with r/r B-ALL. 11 r/r CD19-positive B-ALL patients, aged from 20 to 55 years, who had not previously received CAR-T therapy, signed a written informed consent between December 2018 and June 2020 and were enrolled into the present trial in the Nanjing Drum Tower Hospital. 3 patients failed to receive the CAR-T cell infusion due to disease progression. The remaining 8 patients (4 male and 4 female) received 3 to 20 intensive therapies, completed screening, and were infused with lentiviral-transduced CAR-T cells. Among them, 4 subjects were randomly enrolled in the BBζ CAR-T group, while 4 patients were randomly enrolled in the Dap12-BB group (Table 1).

**Table 1:** Patient characteristics

No.	CAR-T type	Age (years)	Gender	Weight (kg)	Enrolled date	Received treatment	Previous treatment	Dose (cells/kg)	CAR-T positive (%)	Relapse time	Death time	Cri (days)	CR (days)	CRS	CRES
CLH	BBζCAR	21	female	50	2018.12.24	20	VDCLP+MTX&L-Asp+CAM+MA+VDLP+COATD+CTX+PBSCT+MTX+6-MP+hormone+MAE+HyperCAVD&P+MTX&Ara-C+MAED+MTX6.0+VDCP+EA+FAMD+MOAP	1.2 × 10^6^	44.1	M5	M8	30	60	4	0
QM	BBζCAR	23	female	57	2019.05.21	3	VDP+VDCP+HAAG	1.2 × 10^6^	23	–	–	16	45	2	0
YZJ	BBζCAR	27	male	42	2019.05.27	3	VDP+VCP+dasatinib tablets	1.2 × 10^6^	26.4	M4	M7	30	60	1	0
XJJ	BBζCAR	40	male	80	2019.04.01	6	VDCP(3)+Hyper-CAVD(2)+MTX&HD-Ara-C	1.5 × 10^6^	6.3	M3	–	30	60	3	0
LHM	Dap-12 CAR	24	female	42	2019.06.12	3	VDP+VPAP+VDPAP	1.2 × 10^6^	23.7	M14	–	13	30	1	0
LMX	Dap-12 CAR	48	male	62	2019.07.24	7	CRRT+dasatinib tablets(2Y)+IA(2)+VP_16_A+PB&BMSCT+MA+MTX&Ara-C	1.2 × 10^6^	22	M9	–	14	30	0	0
SHW	Dap-12 CAR	20	male	93	2020.05.26	8	VDP(2)+VDCP+VDLP(2)+Ara-C(3)	2 × 10^6^	48	–	–	14	29	3	0
CZL	Dap-12 CAR	55	female	55	2020.06.16	6	IVP+CAM+HD-MTX(2)+IVLP+Hyper-CVAD	2 × 10^6^	43.3	–	–	7	30	1	0

Received treatment means the total number of different therapy or treatment that the patient has received.

VDP, Vincristine + Daunorubicin + Prednison; VPAP, Vincristine + Prednison + Pegaspargase; VDPAP, Vincristine + Daunorubicin + Prednison + Pegaspargase; CRRT, continuous renal replacement therapy; VP-16, Etoposide Ara-C, vincristine + Daunorubicin + Prednison; PB&BMSCT, Peripheral blood & bone marrow stem cells transplantation; MA, Mitoxantrone + Cytarabine; MTX, Methotrexate; VDCP, Vincristine + Daunorubicin + Cyclophosphamide + Prednison; VDCLP, Cyclophosphamide + Vincristine + Daunorubicin + Lasparaginase + Prednisone; L-Asp, Lasparaginase; CAM, Lasparaginase + Cytarabine + 6-mercaptopurine; VDLP, Vincristine + Daunorubicin + Lasparaginase + Prednisone; COATD, Cyclophosphamide + Vindesine + Cytarabine + Tiniposide + dexamethasone; CTX, Cyclophosphamide; PBSCT, Peripheral blood stem cells transplantation; 6-MP, 6-mercaptopurine; MAE, Mitoxantrone + Cytarabine + Etoposide; HyperCAVD, Cyclophosphamide + Vincristine + Doxorubicin + dexamethasone; P, Prednisone; EA, Etoposide + Cytarabine; FAMD, 5-fluorouracil + adriamycin + mitomycin + cisplatin; MOAP, Mitoxantrone + Vincristine + Doxorubicin + Prednisone; HAAG, Recombinant human granulocyte colony stimulating factor + Aclamycin + cytarabine + Harringtonine.

### Safety of CD19-targeted CAR-T cell therapy in adult r/r B-ALL patients

**Figure 4:**
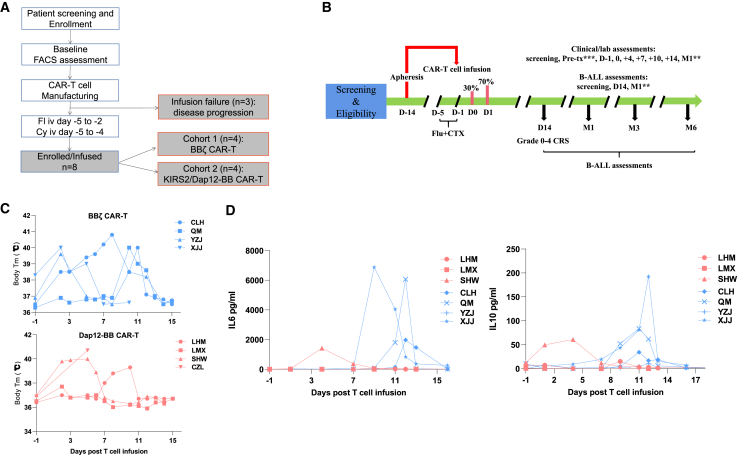
Phase I clinical trial design and the safety of CAR-T cell infusion (A) Schema of the study design and patient enrollment. (B) Treatment schema. CTX, cyclophosphamide. ∗∗After the first 28 days, follow-up is every 4 weeks up to 6 months, then every 3 months up to 2 years. ∗∗∗Pre-tx, pretreatment, 2 to 5 days before CAR-T cell infusion. (C) The line graph shows the patient’s body temperature change after receiving CAR-T cell infusion. (D) IL-6 and IL-10 levels in the subjects’ peripheral blood were detected by ELISA.

All enrolled patients underwent steady-state leukapheresis to collect T cells for CD19 CAR-T manufacturing. CAR-T cells for all the patients were successfully manufactured, achieving the dose of 1–2 × 10^6^ CAR-T cells/kg. Patients received fludarabine and cyclophosphamide-based lymphodepletion chemotherapy consisting of fludarabine (30 mg/m^2^/day, days −5 to −2) and cyclophosphamide (500 mg/m^2^/day, days −5 to −4) before receiving T cell infusion. CD19 CAR-T cells were administered in an outpatient research unit over 2 days as split-dose intravenous infusions (30% [day 0] and 70% [day 1]) (Figures 4A and 4B). The most common adverse event related to CD19 CAR-T cell treatment is the cytokine release syndrome (CRS), and the details are summarized in Table 2. In the BBζ CAR-T group, grade ≥ 3 CRS occurred in 2 patients, while 2 patients experienced grade 2 and 1 CRS. For the patients in the Dap12-BB group, only one patient experienced grade 3 CRS, and 2 patients experienced grade 1 CRS. The main symptoms of CRS are fever and hypotension, and tocilizumab and glucocorticoid were administered to control the CRS once the patient’s temperature was over 38°C (Figure 4C; Table 2). The tocilizumab was administered with 5 mg/dose every 8 h until the patient’s temperature was below 38°C. One patient in each group experienced hypotension, and they were treated with norepinephrine (0.2 μg/kg/min) to promote blood pressure to return to over 80 mm Hg. Moreover, lower IL-6 and IL-10 levels were observed in the patients’ peripheral blood from the Dap12-BB group when compared with that from the BBζ CAR-T group (Figure 4D).

**Table 2:** Summary of adverse events related to CD19-targeted CAR-T cells

All subjects (n = 8)	BBζ CAR-T				KIRS2/Dap12-BB CAR-T			
	Grade 1	Grade 2	Grade 3	Grade 4	Grade 1	Grade 2	Grade 3	Grade 4
CRS	1	1	1	1	2	0	1	0
Fatigue	0	0	0	0	0	0	0	0
Nausea	1	0	0	0	1	0	0	0
Vomiting	6	0	0	0	1	0	0	0
Confusion	0	0	0	0	0	0	0	0
Diarrhea	0	0	0	0	0	0	0	0
Dysgeusia	0	0	0	0	0	0	0	0
Fever	18	10	5	0	4	2	0	0
Abdominal pain	0	0	0	0	1	0	0	0
Anorexia	1	0	0	0	1	0	0	0
Anxiety	0	0	0	0	0	0	0	0
Chills	5	0	0	0	1	0	0	0
Constipation	0	0	0	0	0	0	0	0
Dizziness	1	0	0	0	1	0	0	0
Myalgia	0	0	0	0	0	0	0	0
Paroxysmal atrial tachycardia	1	0	0	0	0	0	0	0
Pleural effusion	1	0	0	0	1	0	0	0
Sore throat	0	0	0	0	0	0	0	0
Abdominal distension	3	2	0	0	0	0	0	0
Acute kidney injury	0	0	0	0	0	0	0	0
Bacteremia	0	0	0	0	0	0	0	0
Hepatic failure	0	0	0	0	0	0	0	0
Hepatitis	0	0	0	0	0	0	0	0
Dyspnea	0	0	0	0	0	0	0	0

**Hematologic events**								

Anemia	0	0	0	0	0	0	0	0
DIC	0	0	0	0	0	0	0	0
Lymphocyte count decreased			2					
Hypotension	0	0	1	0	0	1	0	0

**Nonhematologic events**								

Alkaline phosphatase increased	0	0	0	0	0	0	0	0
ALT increased	0	0	0	0	0	0	0	0
AST increased	1	0	0	0	0	0	0	0
Blood bilirubin increased	0	0	0	0	0	0	0	0

DIC, disseminated intravascular coagulation.

### Bioactivity and clinical response of CD19 CAR-T cells

**Figure 5:**
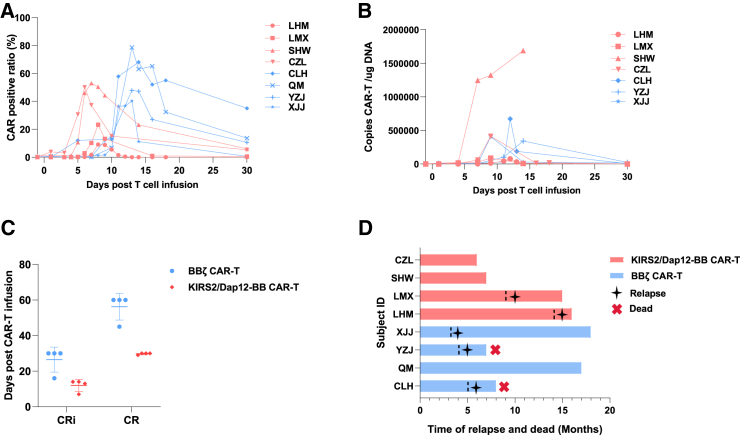
The clinical response of CAR-T cells (A) CAR-T cell expansion and persistence in subjects’ peripheral blood was examined by FACS. (B) CAR-T cell expansion and persistence in subjects’ peripheral blood were examined by qRT-PCR. (C) Dot plot shows the time patients achieved CR. Each dot represents one patient. (D) Swimmer plot shows the time to disease relapse and patient death in months.

The anti-tumor efficacy and clinical response of CAR-T cells are dependent on the proliferative peak and persistence of CAR-T cells *in vivo*. The proliferation and survival of peripheral CD19 CAR-T cells were monitored by fluorescence-activated cell sorting (FACS) and qRT-PCR. In the KIRS2/Dap12-BB group, 4 patients achieved the CAR-T peak at ∼day 7, which is much faster than patients in the BBζ CAR-T group (∼day 14), followed by a decrease with a prolonged follow-up period (Figures 5A and 5B). All 8 patients in two groups achieved complete response (CR) with minimal residual disease negative; even the patient with central nervous system leukemia (CNSL) relapse who received KIRS2/Dap12-BB CAR-T cells also achieved CR, as confirmed by cerebrospinal fluid. Interestingly, patients administered KIRS2/Dap12-BB CAR-T cells achieved CR at ∼day 30, which is much earlier than the patients infused with BBζ CAR-T cells, who ranged from 45 to 60 days (Figure 5C). Although a high CR rate was achieved, 3 of 4 patients infused with BBζ CAR-T cells relapsed in months 3–5, and 2 patients died 2 months later after relapse. Encouragingly, 2 of 4 patients administered KIRS2/Dap12-BB CAR-T cells relapsed in month 9–14, and all 4 patients were still alive, according to the recent follow-up data (Figure 5D). These findings demonstrated that adult r/r B-ALL patients can benefit from the KIRS2/Dap12-BB CAR-T cells.

## Discussion

CAR-T cell therapy is emerging as a promising therapeutic option for hematologic malignancies, with the potential for durable disease control following a single treatment.^3^^,^^19^ We have shown that a KIRS2/DAP12 receptor with scFv triggered antigen-specific cytotoxicity, cytokine production, and proliferation that is comparable with second-generation CD3z-based CARs in a previous study. Incorporation of co-stimulatory domains in CARs has been one approach to enhance anti-tumor efficacy of CAR-T cells, and activation by co-stimulatory domain may confer differential T cell overall therapeutic efficacy. In recent years, co-stimulation receptors derived from 4-1BB or CD28, etc., were incorporated to create second-generation CARs, because the first-generation CAR-T cells engineered with a targeting domain and CD3ζ failed to elicit durable anti-tumor responses.^20^, ^21^, ^22^ It is now clear that incorporation of co-stimulatory signals in CARs is essential to maximize T cell expansion, persistence, and anti-tumor activity. For instance, clinical outcomes from anti-CD19 CAR-T cells demonstrate that inclusion of the CD28 or 4-1BB costimulatory domain enables CAR-T cell long persistence, enhanced cytotoxicity, and a safer profile.^23^, ^24^, ^25^ Thus, costimulatory domains associating with the KIRS2/Dap12 receptor may enhance anti-tumor efficacy of T cells.

This study extends our previous work with the KIRS2/Dap12 receptor and provides a direct comparison of 4-1BB co-stimulation domains in various patterns. We demonstrated that 4-1BB confer KIRS2/Dap12 CAR-expressing T cells with comparable anti-tumor efficacy and more robust cytokine secretion. Importantly, T cells expressing the KIRS2/DAP12-BB demonstrated significant anti-tumor efficacy in xenograft models of B cell lymphoma and pancreatic cancer, which suggests that KIRS2/DAP12-BB targeting CD19 or mesothelin may be preferable for treating hematologic malignancies or mesothelin-positive solid tumors. Furthermore, we evaluated the safety and anti-tumor efficacy of KIRS2/DAP12-BB CAR-T cells in adult r/r B-ALL patients compared with the standard second-generation CD19-targeted CAR-T cells. We successfully manufactured CAR-T cells from all enrolled subjects and observed engraftment in all subjects as well. Interestingly, the peak levels and persistence of CAR-T cells were variable in the two groups. The proliferative peak of KIRS2/DAP12-BB CAR-T in patients’ peripheral blood is at ∼day 7, while BBζ CAR-T achieved peak at ∼day 14. All 8 patients in the two groups achieved CR; however, the patients who received DAP12-BB CAR-T cell (∼30 days) treatment achieved CR faster than patients in the BBζ CAR-T group (range from 45 days to 60 days), which is consistent with their proliferative peak in the peripheral blood after CAR-T cell infusion. One patient with central nervous system leukemia who received DAP12-BB CAR-T treatment also achieved CR, suggesting that DAP12-BB CAR-T cells can migrate into the tumor site and exert an antitumor effect in bone marrow and the central nervous system.

In spite of the promising high CR rate, relapse remains a major problem for CD19 CAR-T cell therapy. Gratifyingly, DAP12-BB CAR-T cell treatment can maintain a longer complete remission, as 2 of 4 patients relapsed on the 14th and 9th month post infusion, while 3 of 4 patients who received BBζ CAR-T treatment relapsed with antigen-positive on 3rd, 4th, and 5th month post infusion and 2 patients died 3 months later. Poor persistence of CAR-T cells is one of the causes for antigen-positive relapse, and murine scFv-derived CAR-T cells may increase the immunogenicity-formed human anti-mouse antibody (HAMA), shorten CAR-T cell persistence, and decrease therapeutic efficiency. These limitations may occur in the present study.^26^, ^27^, ^28^ Additionally, CRS, which is considered as a systemic inflammatory response resulting from CAR-T cell activation and proliferation, is the most common acute toxicity of CAR-T cell therapy.^29^^,^^30^ In our study, all 4 patients in the BBζ CAR-T group experienced the signs and symptoms of CRS, with 2 of 4 patients showing severe CRS (grade ≥ 3), while 3 patients experienced the signs and symptoms of CRS, with 1 of 4 patients showing severe CRS (grade ≥ 3) in the DAP12-BB CAR-T group. Neurotoxicity is another side effect associated with CAR-T cell therapy and is often accompanied by the occurrence of CRS.^31^ However, neurotoxicity does not occur in all patients, even though one patient had central nervous system leukemia. These findings indicate that DAP12-BB CAR-conferred T cells improved safety and enhanced anti-tumor efficacy for adult r/r B-ALL patients.

In summary, autologous T cells expressing CD19 DPK-CAR could expand and induce objective responses with lymphodepleting chemotherapy in subjects with r/r B-ALL and represent a promising new therapeutic approach. The DAP12-BB CAR exerts a lower toxicity profile and enhanced anti-tumor activity in B cell malignancies, given the highly adverse biological features of the enrolled subjects’ B-ALL, including high tumor burden, rapidly progressing disease, and high-risk genetics. Challenges include disease progression during manufacturing, potential for antigen-positive or antigen-negative relapse due to changes in CD19 expression, and durability of responses. Subsequent studies exploring CAR-T manufacturing protocols and off-the-shelf CAR-T products may further optimize the safety and long-term efficacy of this approach.

## Materials and methods

### Generation of CAR constructs

CD19-KIRS2 was designed by splicing a CD19-specific FMC63 scFv antibody onto the transmembrane and short cytoplasmic domain of KIR2DS2. Constructs containing CD19-KIRS2/Dap12, which generated a bicistronic lentiviral vector encoding CD19-KIRS2 and Dap12 separated by the thosea asigna virus 2A (T2A) in combination with the 4-1BB intracellular domain (CD19-KIRS2/Dap12-BB and CD19-KIRS2-BB/Dap12) were generated. The full CAR sequence was synthesized and inserted into the pELPS vector as previously described.^9^^,^^10^

### Cell lines and propagation

Nalm6, AsPC-1, 293T, and MCF7 cells were maintained in a humidified atmosphere containing 5% carbon dioxide (CO_2_) at 37 °C. NALM6 and AsPC-1 cells were cultured in RPMI 1640 (Gibco) medium supplemented with 10% fetal bovine serum (FBS) (Hyclone). The 293T cells were cultured in Dulbecco’s modified Eagle’s medium (DMEM) (Gibco) supplemented with 10% FBS (Hyclone). MCF7-CD19 cells were generated by lentiviral transduction of MCF7 expressing CD19 antigen. All cell lines in this study were assessed by short tandem repeat DNA profiling and routinely examined for mycoplasma contamination by using MycoFluor Mycoplasma Detection kit (Thermo Fisher).

### Lentivirus preparation

Lentivirus supernatant used for the transduction of human T cells was prepared using a non-replicative and self-inactivating third-generation system. Briefly, 1 × 10^7^ HEK293T cells (ATCC) cultured in T150 flask (Corning) were transfected with the packaging plasmids pRSV-Rev, pMDLg-pRRE, and pVSV-G and the transfer plasmid pELPS containing the CAR using empty DMEM (Gibco) medium and PEI transfection reagent (Polysciences). 48 h later, supernatant containing lentivirus particles was collected and concentrated by ultracentrifugation. Viral titer in TU/mL was determined by serial dilution and infection of HEK293T cells.

### CD19 CAR-T cell manufacturing

Peripheral blood mononuclear cells (PBMCs) were collected from healthy donors, and CD3+ T cells were separated and stimulated with CD3/CD28 Dynabeads. CD3+ T cells were cultured in X-VIVO 15 media (Lonza) with 300 U/mL IL-2 and 5% autologous plasma. Then, the T cells were transduced with CAR lentivirus. Transduction efficiency and cell viability were examined at 5 and 8 days, and the medium was replaced every day. Subsequently, the percentage of CAR+ T cells was determined by flow cytometry using anti-mouse FMC63 scFv monoclonal antibody (Bioswan). On day 8–12, T cells are collected for *in vitro* and *in vivo* functional assays. Quality controls of CAR-T cells for fungi, bacteria, mycoplasma, chlamydia, and endotoxin were performed before cell infusion.

### Generation of clinical grade CD19 CAR-T cells

Fresh PBMCs collected from patients at day 0, and CD3+ T cells, were separated and stimulated with CD3/CD28 Dynabeads at a 1:3 cell-to-bead ratio in X-VIVO 15 medium supplemented with 5% autologous plasma, 2 mmol/L L-glutamine and 300 U/mL of recombinant human IL-2 at 0.5 × 106 cells/mL. Activated T cells were transduced with CD19 CAR lentiviral supernatant in 6-well plates at 0.5 × 106 cells/mL in the presence of 300 U/mL of rhIL-2 at day 1. Lentivirus was removed by centrifugation after 48 h transduction, and CAR-T cells were then expanded in culture flasks until scaled up to cell culture bags in complete X-VIVO 15 medium supplemented with 300 U/mL of rhIL-2 for 10–12 days. On the day of harvest, CAR-T cells were formulated in CS250 cryostorage bags to achieve the target dose in CryoStor CS10. Quality controls of CAR-T cells for fungi, bacteria, mycoplasma, chlamydia, and endotoxin were performed before cell infusion.

### Real-time qPCR

The total RNA of NTD, KIRS2/Dap12-BB CAR-T cells, and 4-1BB/CD3ζ CAR-T cells stimulated with CD19-MCF7 cells were isolated using the FastPure Cell/Tissue Total RNA Isolation Kit V2 (Vazyme, Nanjing, China) according to the manufacturer’s instructions. Next, 1 μg of purified RNA was reverse transcribed into cDNA by using HiScript III RT SuperMix for qPCR (+gDNA wiper) (Vazyme, Nanjing, China) according to the manufacturer’s instructions. Then, the qPCR analysis was conducted on the Applied Biosystems 7500 Real-Time PCR Systems using ChamQ SYBR qPCR Master Mix (Vazyme, Nanjing, China) according to the manufacturer’s instructions. The housekeeping gene glyceraldehyde-3-phosphate dehydrogenase (GAPDH) was used as an internal control, and the qPCR data were then analyzed with relative threshold cycle (CT) values and converted to fold changes normalized to control cells. The sequences of primers used in this study are listed in Table S1.

### Mouse xenograft models

Xenograft models were performed as previously described. Briefly, 6- to 7-week-old NOD-*Prkdc*^*em26Cd52*^*Il2rg*^*em26Cd22*^ mice were obtained from the model animal research center of Nanjing University and maintained under pathogen-free conditions. Animals were injected with 5 × 10^5^ Nalm6 cells or 2 × 10^6^ AsPC-1 cells subcutaneously (s.c.) in a volume of 0.1 mL. The mice with tumor burden were infused with CAR-T cells via tail intravenous injection on days 15 and 21 following Nalm6 and AsPC-1 injection, respectively. Mice were closely monitored for signs of graft-versus-host disease as evidenced by >10% weight loss, loss of fur and*/*or diarrhea, and tumor volume. All protocols were approved by the Committee on the Ethics of Animal Experiments of the Nanjing University and were strictly carried out in accordance with the Guide for the Care and Use of Laboratory Animals of the National Institutes of Health.

### Flow cytometry

Expression of the various CD19-CARs on T cells was detected using either biotinylated anti-mouse FMC63 scFv monoclonal antibody (Biowan) followed by staining with streptavidin-phycoerythrin (PE) (BD Biosciences), or with an allophycocyanin (APC)-labeled anti-mouse FMC63 scFv monoclonal antibody (Biowan). 1 × 10^6^ cells were stained for cell surface markers to analyze T cell differentiation and exhaust status. The following antibodies were used: anti-CD3-FITC (BD Biosciences), anti-human CD3-APC (BD Biosciences), anti-CCR7-BV421 (BD Biosciences), anti-CD 45RO-APC (BD Biosciences), anti-CD4-BB515, anti-CD8-BV510 (BD Biosciences), anti-CD45RA-PE- Cy7 (BD Biosciences), anti-CD62L-PE (BD Biosciences), anti-PD-1-APC (BD Biosciences), anti-TIM-3APC (BD Biosciences), and anti-LAG-3-APC (BD Biosciences). Samples were analyzed on either FACSCanto II flow cytometers (BD Biosciences) or Novocyte3110 (AECE).

### Proliferation and cytokine secretion

T cell proliferation ability was assessed with a carboxyfluorescein diacetate succinimidyl ester (CFSE) dilution assay. T cells were washed and stained with 5 μM CFSE (BD Biosciences) for 10 min at 37°C. The reaction was quenched by adding PBS with 10% FBS and washed twice with the same buffer. CAR-T cells were then incubated only or co-incubated at a ratio of 1:1 with target cells for 5 days. Supernatants were also collected at 24 h to assess cytokine production. Measurement of cytokine was performed using IL-2, IFN-γ, and TNF-ɑ ELISA kits (R&D) according to the manufacturer’s instructions. All samples were analyzed in triplicate and compared against multiple internal standards with a seven-point standard curve.

### Cytotoxicity assays

Cytotoxicity was assessed using the xCELLigence Real-Time Cell Analyzer (RTCA). MCF7 and MCF7-CD19 cells (1 × 10^4^ cells per well) were added to an E-Plate. 18 h later, CAR-T cells were added to the E-Plate according to different E:T ratios (E:T = 0:1, 1:1, 5:1, 10:1). The normalized cell index was recorded every 15 min and monitored for at least 20 h. The specific lysis rate was calculated according to the formula:

$$\%cytolysis=\frac{CI\left(No-Effector\right)-CI\left(Effector\right)}{CI\left(No-Effector\right)}.$$

### Phase I study design and participants

We conducted a phase I study (ChiCTR1800016584) designed to assess the bioactivity and safety of CD19-KIRS2/Dap12-BB CAR-T cells compared with standard second-generation CD19-CD3/4-1BB CAR-T cells. Autologous T cells from the patients with r/r CD19+ B cell malignant tumor (B-ALL and non-Hodgkin lymphoma) were genetically modified to express a CD19-specific CAR encoding the KIRS2/DAP12-BB or 4-1BB/CD3ζ. Patients with relapsed and/or refractory CD19-positive B-ALL were enrolled in this phase I study as eligible subjects restricting to the key eligibility criteria, including age ≤ 70 years, KPS (Karnofsky performance score) ≥ 60, serum creatinine no greater than 200 μmol/L, absolute platelet count at least 75 × 10^9^/L and lymphocyte count at least 0.15 × 10^9^/L, total bilirubin no greater than 30 μmol, ALT no greater than 100 U/L and AST no greater than 100 U/L, lack of active autoimmune disease, and creatinine less than 200 mol/L. Exclusion criteria included central nervous system leukemia (symptoms, signs, imaging, cerebrospinal fluid) can be found; patients with hyperleukemia (white blood cell count > 50 × 10^9^/L) or patients who were able to judge the progression of the disease quickly when they were enrolled and unable to ensure a complete cycle of treatment; patients with infection including fungi, bacteria, viruses, or other uncontrollable infections or grade four isolation treatment; HIV-, HBV-, and HCV-positive patients; patients with central nervous system diseases or autoimmune central nervous system diseases, including stroke, epilepsy, dementia, etc.; in the first 12 months of the study were associated with myocardial infection, cardiac angiography or stent, active angina or other obvious clinical symptoms, or associated with coronary heart disease or cardiovascular lymphocytic infiltration; receiving anticoagulant therapy or severe coagulation disorders (activated partial thromboplastin time > 70); according to the researchers’ judgement, the drug treatment that the patient is receiving will affect the safety and effectiveness of the project; patients with allergy or allergy to biological agents used in this project; pregnant or lactating women; within 2 weeks before treatment, systemic and systemic steroid medications were used (except for inhaled steroids recently or currently being used); suffering from other uncontrolled diseases the researchers consider inappropriate for participants; researchers believe that any condition that may increase the risk or interfere with the outcome of the test may be increased; participated in other clinical studies. All patients enrolled in this study provided signed written informed consent, and this study was approved by the review board of Nanjing Drum Tower Hospital.

All patients were screened based on the inclusion and exclusion criteria before collection of PBMCs that were used for CAR T cell manufacturing. Patients were enrolled into two cohorts to receive 1.2–2 × 10^6^ cells/kg CD19 BBζ CAR-T cells (cohort 1, n = 4) or KIRS2/Dap12-BB CAR-T cells (cohort 2, n = 4) with lymphodepletion using 500 mg/m^2^ cyclophosphamide administered intravenously 2 days (day −5 and day −4) and 30 mg/m^2^ fludarabine administered intravenously 4 days (day −5, −4, −3, and 2) prior to CD19 CAR-T cells infusion. Dose escalation was not conducted in this study. Peripheral blood from patients were collected at defined time points to monitor the CAR-T cells’ persistence, safety, and efficacy. Safety assessments included monitoring the treatment-related adverse events (AEs) according to National Cancer Institute (NCI) Common Terminology Criteria of AE version 4.0. Study-related AEs included infusional toxicity and any toxicity that possibly associated with CD19 CAR-T cells, such as CRS, CRES, and hypogammaglobulinemia etc. To evaluate the therapeutic effect of CAR-T cells, follow-ups were performed on the 2nd, 7th, 14th and 28th days after the last infusion. From the 4th week to 6 months, follow-ups were performed on the 28th, 56th, 90th, and 180th days; until the 2nd year, follow-ups were performed every 8 weeks.

### Cytometric bead array for patient IL-6 assay

We analyzed plasma or serum samples from enrolled patients collected before and after CD19 CAR-T cell infusion using a BD cytokine cytometric bead array (CBA) kit (Becton Dickinson/Pharmingen), following the manufacturer’s instructions. In parallel with the samples, we used the human cytokine standard samples provided with the kit to prepare standard curves. We performed the CBA assays using the FACSCanto II (Becton Dickinson).

### Ethics statement

Research involving human materials was approved by Ethical Committee of Nanjing Drum Tower Hospital. Peripheral blood (PB) was obtained from healthy donors or patients after informed consent.

### Statistical analysis

The statistical analysis of *in vivo* and *in vitro* experimental data in this study was conducted on SPSS 18.0 (IBM, Armonk, NY, USA) and GraphPad Prism 8.0 software. Results of the analysis were represented as mean ± SD. The statistical significance between different groups was assessed by using Student’s t-test, and a p value equal to or less than 0.05 was defined as significant.

### Ethics approval and consent to participate

The study was reviewed and approved by Nanjing Drum Tower Hospital and Children’s Hospital of Nanjing Medical University. The regulatory sponsor was Nanjing CART Medical Science and Technology. All subjects provided written informed consent prior to participation.

### Availability of data and materials

The dataset(s) supporting the findings of this study are included within the article and available from the corresponding author on reasonable request.

## Supplemental information

Document S1. Table S1 and Figure S1

Document S2. Article plus supplemental information
