# Supplementary material for: Novel two-chain structure utilizing KIRS2/DAP12 domain improves the safety and efficacy of CAR-T cells in adults with r/r B-ALL
Source: Mol Ther Oncolytics. 2021 Aug 28;23:96–106. doi: 10.1016/j.omto.2021.08.014 (PMC8517091; doi:10.1016/j.omto.2021.08.014)
Supplement: Document S1. Table S1 and Figure S1 [file mmc1.pdf]

**OMTO, Volume *23***

## Supplemental information

# Novel two-chain structure utilizing KIRS2/DAP12 domain improves the safety and efficacy of CAR-T cells in adults with r/r B-ALL

**Ming Sun, Peipei Xu, Enxiu Wang, Min Zhou, Tongpeng Xu, Jing Wang, Qian Wang, Bo Wang, Kaihua Lu, Chen Wang, and Bing Chen**

**Supplementary Table1.** The sequence of primers used in this study.

| Gene | Sequence |
| --- | --- |
| GM-CSF(CSF2) F | TCCTGAACCTGAGTAGAGACAC |
| GM-CSF(CSF2) R | TGCTGCTTGTAGTGGCTGG |
| IL-10 F | GACTTTAAGGGTTACCTGGGTTG |
| IL-10 R | TCACATGCGCCTTGATGTCTG |
| IL-12A F | ATGGCCCTGTGCCTTAGTAGT |
| IL-12A R | AGCTTTGCATTCATGGTCTTGA |
| IL-2 F | AACTCCTGTCTTGCATTGCAC |
| IL-2 R | GCTCCAGTTGTAGCTGTGTTT |
| IL-6 F | CCTGAACCTTCCAAAGATGGC |
| IL-6 R | TTCACCAGGCAAGTCTCCTCA |
| IFNG F | TCGGTAACTGACTTGAATGTCCA |
| IFNG R | TCGCTTCCCTGTTTTAGCTGC |
| TNFA F | GAGGCCAAGCCCTGGTATG |
| TNFA R | CGGGCCGATTGATCTCAGC |
| GZMB F | CCCTGGGAAAACACTCACACA |
| GZMB R | GCACAACTCAATGGTACTGTCG |
| PRF1 F | GACTGCCTGACTGTCGAGG |
| PRF1 R | TCCCGGTAGGTTTGGTGGAA |

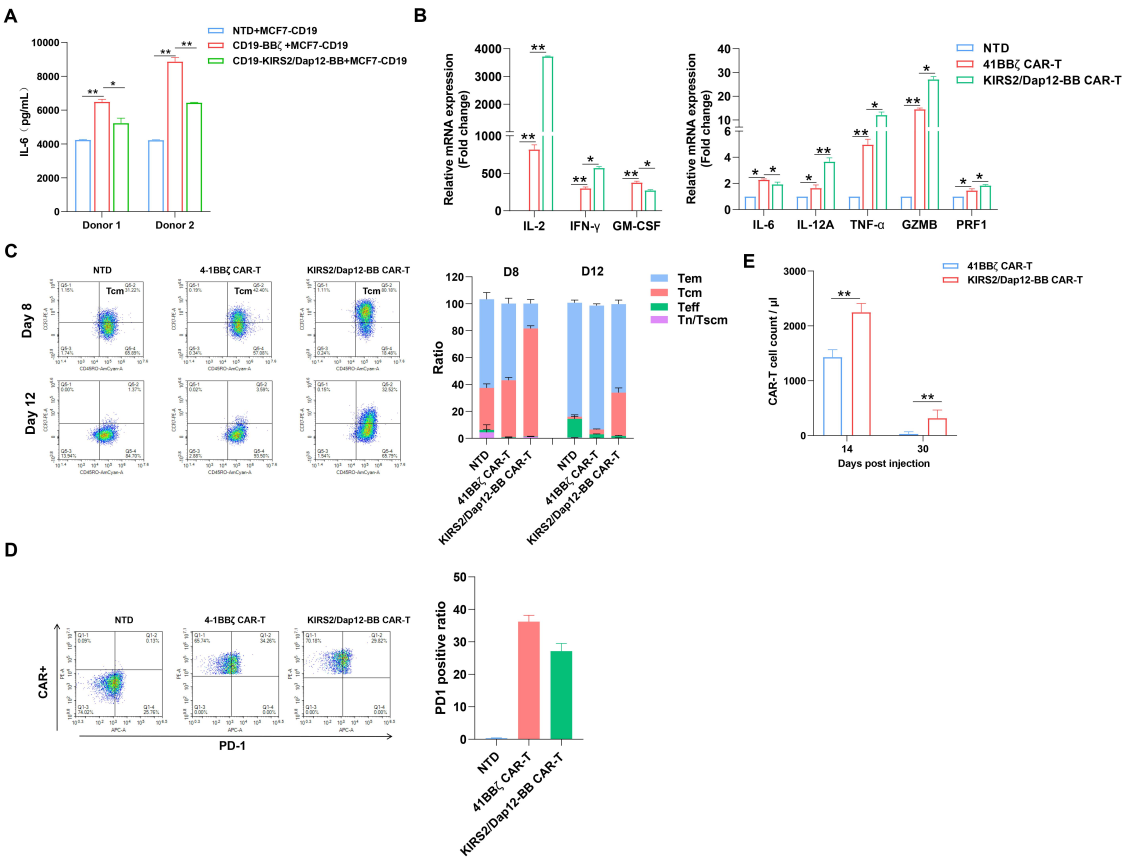


**Supplementary Figure1. Relative expression of cytokine, checkpoint receptors and memory T cell subsets in CD19 targeted CAR-T cells.** (A) IL-6 released in the culture supernatant by NTD, and CAR-T cells was measured by ELISA (n = 3). (B) qPCR analysis of IL-2, IL-6, IL-12A, IFN-γ, TNF-α, GM-CSF, GZMB and PRF1 in NTD, KIRS2/Dap12-BB and BBζ CAR-T cells. (C) Gating strategy used to identify Tn, Tcm, Tem and Teff subsets of NTD, KIRS2/Dap12-BB and BBζ CAR-T cells by flow cytometry. (D) Flow cytometry analysis of T cell surface exhaustion marker PD-1 expression in NTD, KIRS2/Dap12-BB and BBζ CAR-T cells on Day8 and Day12 after T cell activation. (E) Flow cytometry analysis of CD19 CAR-T cells in mice tail vein blood from NTD, KIRS2/Dap12-BB and BBζ CAR-T cells groups. * $p<0.05$, ** $p<0.01$
